# Prevalence and Risk Factors for Oral Potentially Malignant Disorders in Indian Population

**DOI:** 10.1155/2015/208519

**Published:** 2015-08-11

**Authors:** Sandeep Kumar, Nitai Debnath, Mohammed B. Ismail, Arunoday Kumar, Amit Kumar, Bhumika K. Badiyani, Pavan K. Dubey, Laxmi V. Sukhtankar

**Affiliations:** ^1^Department of Public Health Dentistry, Sri Aurobindo College of Dentistry, Indore, Madhya Pradesh 453555, India; ^2^Department of Prosthodontics, Dental College, RIMS, Imphal, Manipur 795004, India; ^3^Department of Periodontics, GDC-RI, VIMS, Bellary, Karnataka 583102, India; ^4^Department of Prosthodontics, Hazaribag College of Dental Sciences, Jharkhand 825301, India; ^5^Department of Public Health Dentistry, Sarjug Dental College and Hospital, Bihar 846003, India; ^6^Prosthodontics, Dental Evolution Clinic, Varanasi, Uttar Pradesh 221002, India; ^7^Periodontics, Troy, MI, USA

## Abstract

*Objective*. To assess the prevalence of oral potentially malignant disorders and to determine the potential risk factors for its development in Indian population. *Materials and Methods*. This cross-sectional study was carried out on 1241 individuals in Indore, Madhya Pradesh. A questionnaire was designed to record information about sociodemographic characteristics, oral hygiene practices, dietary habits, and risk factors for oral potentially malignant disorders. Oral mucosal lesions were examined by a skilled person. *Results*. The overall prevalence of oral potentially malignant disorders was found to be 13.7% with oral submucous fibrosis (8.06%) found to be more common and erythroplakia (0.24%) found to be least prevalent. Results of Logistic Regression analysis showed that males (OR = 2.09, *P* value < 0.0001) who were ever consumers of tobacco (OR = 2.06, *P* value = 0.030) and areca nut chewing (OR = 2.64, *P* value = 0.004) were more likely to develop oral potentially malignant disorders compared to never consumers. Diabetic (OR = 2.21, *P* value = 0.014) and underweight individuals (OR = 2.23, *P* value = 0.007) were more likely to suffer from oral potentially malignant disorders. *Conclusion*. The study reinforces the association of tobacco and areca nut consumption with oral potentially malignant disorders. An association of oral potentially malignant disorders with diabetes and BMI was confirmed by this study.

## 1. Introduction

Oral cancer is a serious and growing problem in many parts of the globe. Oral and pharyngeal cancer, grouped together, are the sixth most common cancer in the world [1]. Oral cancer is sometimes preceded by clinically visible lesions which are noncancerous to begin with and which have therefore been termed precancerous. The most common oral potentially malignant lesions are leukoplakia, erythroplakia, and oral submucous fibrosis. A large number of these oral mucosal lesions have a tendency to transform into malignancy. The malignant transformations of oral mucosal lesions including leukoplakia [2], erythroplakia [3], and submucous fibrosis [4] are well documented.

Tobacco has been considered as a major etiological factor in the development of oral potentially malignant disorders. A variety of oral potentially malignant disorders have been reported in literature with the consumption of tobacco [5, 6]. In Asians, oral potentially malignant disorders are known to be associated with cigarette smoking, excess alcohol consumption, and areca quid chewing [7]. Besides these, diabetes [8], body mass index [5], and certain dietary factors like low vegetable intake and less frequency of fruits consumption are independent risk factors for development of oral potentially malignant disorders [9].

Oral potentially malignant disorder and its sequelae may cause heavy impairment in quality of life; the disease is also highly costly for society. Primary prevention is the most cost effective prevention program as it aims to reduce the incidence of potentially malignant disorders, by risk factor modification. Most of the general public is poorly informed about the risk of oral potentially malignant disorder and ways to prevent this disease. Early detection is of critical importance, and survival rates markedly improve when identified at early stage. Investigating the prevalence of oral mucosal lesions will prevent malignant transformation. With limited literature available to draw conclusions about the prevalence of oral potentially malignant disorders, more studies are needed in order to better understand the epidemiology of this destructive disorder. Hence, this study was performed to assess the prevalence of oral potentially malignant disorders and to determine the potential risk factors for its development in an Indian population.

## 2. Materials and Methods

A cross-sectional study was conducted in Indore district of Madhya Pradesh. This was a household survey conducted in the months of March–June 2014 for about 4 months. The study comprised 1241 inhabitants of Indore district. The district was divided into four zones for the study purpose and a stratified cluster sampling design was employed in which a random sample of 200 households was selected from each of the four strata and individuals were randomly selected from each household. A pilot study was conducted on 25 randomly picked individuals. The participants of the pilot study were not a part of the main survey. Pilot survey assessments were utilized for sample size estimation and proper planning and execution of the main study. In accordance with the ADA classification (1970) type 3 method of examination using a disposable mouth mirror (Patterson) and explorer was carried out under good illumination. The patients were seated on chairs in their houses and were examined. The final sample size was calculated using the formula for sample size calculation recommended by W.H.O. [10]. Consider

(1) $$\begin{array}{c} N=\frac{z^{2}p\left(1-p\right)}{d^{2}}, \end{array}$$

where *z* = 1.96 for 95% confidence interval, *p* = prevalence of disease in a population, and *d* = acceptable margin of error (0.05).

A prevalence of 30% of oral potentially malignant disorders was found in the population when the pilot study was conducted. Substituting the values in the above formula, it was found that a minimal sample size of 323 would be required for the study. The study however included a larger sample in order to improve its precision.

The ethical approval to conduct the study was taken from the Institutional Research committee of Sri Aurobindo Institute of Medical Sciences. A leaflet of study objectives accompanied with an informed consent was delivered to the participants. A total of 656 males and 585 females in the age group of 20–65 years participated in the survey. The study objectives were explained to the participants before commencing with the interview. The inclusion criteria consisted of age above 18 years and willingness to participate in oral examination along with a written consent filled in. The exclusion criteria consisted of all individuals below 18 years of age and not being willing to undergo oral examination and not giving consent or suffering from any systemic disease. The participants had the right to withdraw at any point of time from the study and no incentives were given to increase participation.

A questionnaire was prepared which comprised five sections. The first part of the questionnaire collected information on sociodemographic characteristics like age, gender, and socioeconomic status. Socioeconomic status was calculated using modified Kuppuswamy scale [11] and categorized into three strata of upper, middle, and lower class.

The second part of the questionnaire dealt with oral hygiene practices of the participants. The third part of the questionnaire collected information on adverse habit like tobacco chewing, pan chewing, and areca nut chewing and alcohol consumption. The responses were dichotomized into ever consumers and never consumers. The fourth part of the questionnaire dealt with dietary risk factors like frequency of vegetable intake and frequency of fruits intake. The dietary risk factors were evaluated on a six-point Likert scale: seldom/never, several times a month, once a week, several times a week, every day, and several times in a day. In addition, the questionnaire collected information on dental visit, history of diabetes, wearing of dental prosthesis, and use of mouthwash. The responses were categorized into Yes and No. The questionnaire was translated into local language. The validity was checked by a back translational method involving a blind retranslation into English. Pretesting of the questionnaire was done and substantial wording modifications were made. Subject experts in both the languages verified the validity of the questionnaire after translation. The duplicate examination was carried out after 2-week interval on 50 participants to assess the intrarater reliability.

A face to face interview was conducted by a trained and calibrated examiner. The training and calibration were performed in Sri Aurobindo Dental College. The investigator was trained to diagnose the commonly occurring oral potentially malignant lesions like oral submucous fibrosis, lichen planus, leukoplakia, and erythroplakia using standardized criteria [12]. Face to face interview method was selected in order to avoid incomplete submission. Any doubts arising during the filling of the questionnaire were clarified by the interviewer. An oral examination was conducted and the entire oral mucosa was checked for signs of oral potentially malignant disorders using mouth mirror. To avoid bias, data collection and oral examination were performed by the same expert.

Following this, the body mass index was calculated using the formulae BMI [13] = height/weight^2^. An intern who was trained for the purpose carried out the anthropometric measurements. The weight of an individual was determined using weighing machine (NOVA) and height was determined using a stadiometer (SCA217, PORTABLE, 8–81′′). It was then categorized into three strata underweight, normal, and overweight/obese based upon International Classification of Obesity.

All statistical analysis was performed using SPSS version 16.5. Chi Square test was performed for categorical data. Logistic Regression analysis was performed to identify the predictors for potentially malignant disorders. *P* value < 0.05 was considered statistically significant.

## 3. Results

The study population comprised 52.9% males and 47.1% females. A larger proportion of the study population belonged to either middle class (32.5%) or lower class (40.5%). The majority (62.9%) of them used toothbrush for oral hygiene maintenance (Table 1).

13.7% of the Indian population showed the presence of oral potentially malignant disorders. Oral submucous fibrosis was found in 8.06% of the population, leukoplakia in 4.02% of the population, and Lichen planus in 1.38% of the population. The least prevalent oral potentially malignant disorders were erythroplakia (0.24%) (Table 2).

Males (17.4%) were found to have a significantly higher prevalence (*P*  value < 0.0001^*∗*^) of oral potentially malignant disorders compared to females. No significant differences were found between different categories of socioeconomic status, tooth brushing methods, and brushing frequency with prevalence of oral potentially malignant disorders.

A significantly higher prevalence (*P*  value < 0.0001) of oral potentially malignant disorders was found in individuals who were ever consumers of areca nut chewing (32.9%) and tobacco consumption (28.4%) compared to never consumers. No significant differences were observed between various categories of alcohol consumption and betel quid consumption with prevalence of oral potentially malignant disorders (Table 3).

A significantly higher prevalence of oral potentially malignant disorders was found in individuals who had visited dentist and were wearers of dental prosthesis (*P*  value < 0.0001). Diabetic patients (24.6%) and underweight individuals (20.1%) showed a higher prevalence of oral potentially malignant disorders (*P*  value < 0.0001) (Table 4).

Results of Logistic Regression analysis showed that males (OR = 2.09, *P*  value < 0.0001) were more likely to have oral potentially malignant disorders compared to females. The individuals who were ever consumers of tobacco (OR = 2.06, *P*  value = 0.030) and areca nut chewing (OR = 2.64, *P*  value = 0.004) were more likely to develop oral potentially malignant disorders compared to never users. Diabetic individuals (OR = 2.21, *P*  value = 0.014) were more likely to suffer from oral potentially malignant disorders compared to nondiabetics. An inverse relationship was observed between body mass index (BMI) and development of oral potentially malignant disorders wherein underweight individuals (OR = 2.23, *P*  value = 0.007) showed higher tendency to develop oral potentially malignant disorders (Table 5).

## 4. Discussion

This study was conducted with the objective of assessing the prevalence of oral potentially malignant disorder and of determining the potential risk factors for its development in Indian population. Data were collected using a pretested questionnaire by a trained and calibrated person. The strength of the study was that the study took into consideration a larger sample size than was required for the study which helped to improve the precision of the study findings. Also the study populations were recruited using a house to house survey based on random sampling method which provided more accurate data and better representation from all sections of the society. The face to face interview helped to minimize incomplete submission and clarification of the questionnaire was done on the spot if any difficulties were encountered.

The results of this study showed that the overall prevalence of oral potentially malignant disorders in the study population was 13.7%. Oral submucous fibrosis (8.06%) was most commonly seen and erythroplakia was least prevalent (0.24%). Similar findings and similar spectrum of distribution of oral potentially malignant disorders were detected in Taiwan in a study conducted by Chung et al. [7]. In a recent screening program conducted in India by Warnakulasuriya et al. [14] to detect potentially malignant oral disorders within a workplace, a similar prevalence (5%) of leukoplakia was found. Oral submucous fibrosis showed the highest prevalence (8.06%) amongst the diagnosed oral potentially malignant disorders which were similar to the findings reported by Mehrotra et al. [15]. However, the prevalence of oral potentially malignant disorders has a varied distribution globally as reported by various studies [7]. This may be attributed to difference in tobacco consumption and cultural, dietary, and environmental factors.

In the present study, gender was found to be significantly associated with development of oral potentially malignant disorders with males being at higher risk to develop oral potentially malignant disorders. In a review done by Nair et al. [16], the prevalence of oral potentially malignant disorders and oral cancer was found to be more in males. A similar finding was reported in study conducted by Chung et al. [7], in Taiwan, wherein a statistical significant difference was observed between various oral potentially malignant disorders detected and gender. A reason that the authors believe for this gender discrepancy with males being at higher risk may be due to the fact that the habit of tobacco consumption is more in males which may lead to development of oral potentially malignant disorders in males.

Various studies have reported a strong association between tobacco consumption and development of oral potentially malignant disorders [17]. In the present study also, tobacco was found as a strong predictor for development of oral potentially malignant disorders. More than 60 known carcinogens have been detected in tobacco [18]. This chemical after coming in contact with oral mucosa accelerates inflammatory process and on long term abuse is responsible for atrophic and hypertrophic changes in the oral mucosa.

Areca nut chewing has long been associated with the development of oral potentially malignant disorders. Various studies conducted in different parts of the world have reported adverse effects of areca nut consumption [7, 17]. Some areca nut specific nitrosamines suspected to be carcinogenic are 3-methylnitrosamino propionaldehyde (MNPA), 3-methylnitrosamino propionitrile (MNPN), N-nitrosoguvacine (NGC), and N-nitrosoguvacoline (NGL). MNPA in particular causes DNA single strand breaks and DNA protein cross-links [19].

Interestingly, the results of this study showed that individuals who had visited a dentist have higher prevalence of oral potentially malignant disorders than individuals who had never visited a dentist. The results are consistent with the findings of Mtaya et al. [20] and Masalu and Åstrøm [21]. This may be attributed to health care seeking behavior of the respondents. The people seek dental care only when there is a severe oral condition which warrants urgent oral care. Okunseri et al. [22] have termed this type of phenomenon as “healthy person nonvisitor effect.” However, when Logistic Regression analysis was performed no significant differences were observed between the two categories.

Diabetes is a chronic disorder known to affect oral disease progression [23]. Various studies have found a positive association between diabetes and development of oral potentially malignant disorders [24, 25]. In the present study also, diabetes was found to be a significant risk factor for the development of oral potentially malignant disorders. A diabetic individual has poor oral hygiene and suffers from hyposalivation and xerostomia which might predispose them to increased susceptibility to infections and to development of oral mucosal diseases [26].

In the present study an inverse relationship was observed between body mass index and development of oral potentially malignant disorders. The underweight individuals were more likely to develop oral potentially malignant disorders in agreement with previous studies conducted by Kabat et al. [27] and Thomas et al. [5]. Lower BMI may be associated with lower socioeconomic status and malnutrition due to lower socioeconomic status [28]. Hormonal imbalance and mucosal atrophy are other risk factors increasing the risk for oral potentially malignant disorders to develop in underweight individuals [29]. Besides these some studies have reported an inverse association of BMI with the development of oral potentially malignant disorders due to immune dysfunction and hormonal imbalance [30].

Results of Logistic Regression analysis showed that gender, tobacco consumption, areca nut chewing, body mass index, and diabetes were best predictors for development of oral potentially malignant disorders in the study population.

The study has certain limitations. The diagnosis of oral potentially malignant disorders was not confirmed by taking biopsy. Also, as many of the individuals have mixed habits of tobacco chewing and areca nut chewing therefore the exact role of these individual risk factors is difficult to predict. A possible limitation of the study may be social desirability bias wherein males and females participating in the survey might have underreported frequency of tobacco and alcohol consumption to the interviewer due to social expectations. Another limitation of this study is the cross-sectional nature of the study design so the temporal relationship of the oral potentially malignant disorders and its associated risk factors comes into question. Finally, we also acknowledge that there might be other risk factors that may be responsible for development of oral potentially malignant disorders which we have not taken into consideration, so further research is warranted.

## 5. Conclusion

The oral potentially malignant disorders have tendency to transform into malignancy. The overall prevalence of oral potentially malignant disorders was found to be 13.7% with oral submucous fibrosis to be more predominant in the Indian population. The consumption of tobacco and consumption of areca nut were identified as risk factors for development of oral potentially malignant disorders. Diabetic and underweight individuals were found to be at increased risk. The identification of these risk factors allows for better preventive efforts against this disorder. Individuals who have already developed this disorder may be advised to reduce the exposure to this risk factor which may prevent further progression of this disorder. Appropriate steps should be taken for early intervention, as it is the key to effective prevention.

## Figures and Tables

**Table 1:** Socio demographic characteristics and oral hygiene practices of the respondents.

Characteristics	Categories	Number (%)
Gender	Male	656 (52.9%)
	Female	585 (47.1%)

Socioeconomic status	Upper class	336 (27.1%)
	Middle class	403 (32.5%)
	Lower class	502 (40.5%)

Tooth brushing method	Toothbrush	780 (62.9%)
	Finger	221 (17.8%)
	Other methods	240 (19.3%)

Brushing frequency	Once/daily	908 (73.2%)
	Twice/daily	210 (16.9%)
	More than twice/daily	123 (9.9%)

**Table 2:** Prevalence of oral potentially malignant disorders in the study population.

Prevalence	Number (%)
Oral sub mucous fibrosis	100 (8.06%)
Leukoplakia	50 (4.02%)
Lichen Planus	17 (1.38%)
Erythroplakia	3 (0.24%)
Total	170 (13.7%)

**Table 3:** Association of socio demographic characteristics, oral hygiene practices and adverse oral habits with oral potentially malignant disorders.

Factors	Categories	Oral potentially malignant disorder present *N* (%)	Oral potentially malignant disorder absent *N* (%)	*p* value
Gender	Male	114 (17.4%)	542 (82.6%)	<0.0001^*∗*^
	Female	56 (9.6%)	529 (90.4%0	

Socioeconomic status	Upper class	50 (14.9%)	286 (85.1%)	0.337
	Middle class	60 (14.9%)	343 (85.1%)	
	Lower class	60 (12%)	442 (88.0%)	

Toothbrushing method	Toothbrush	100 (12.8)	680 (87.2%)	0.461
	Finger	32 (14.5%)	189 (85.5%)	
	Other methods	38 (15.8%)	202 (84.2%)	

Brushing frequency	Once/daily	115 (12.7%)	793 (87.3%)	0.146
	Twice/daily	32 (15.2%)	178 (84.8%)	
	More than twice/daily	23 (18.7%)	100 (81.3%)	

Alcohol consumption	Ever consumers	33 (18.2%)	148 (81.8%)	0.061
	Never consumers	137 (12.9%)	923 (87.1%)	

Betel quid consumption	Ever consumers	34 (16.9%)	167 (83.1%)	0.147
	Never consumers	136 (13.1%)	904 (86.9%)	

Tobacco consumption	Ever consumers	94 (28.4%)	237 (71.6%)	<0.0001^*∗*^
	Never consumers	76 (8.4%)	834 (91.6%)	

Areca nut chewing	Ever consumers	82 (32.9)	167 (67.1%)	<0.0001^*∗*^
	Never consumers	88 (8.9%)	904 (91.1%)	

^*∗*^ *p* value < 0.05: statistical significant difference.

**Table 4:** Association of dietary and other risk factors with oral potentially malignant disorders.

Factors	Categories	Oral potentially malignant disorder present *N* (%)	Oral potentially malignant disorder absent *N* (%)	*p* value
Frequency of fruits consumption	Several times a day	38 (13.0%)	255 (87.0%)	0.651
	Everyday	51 (14.5%)	301 (85.5%)	
	Several times a week	55 (12.3%)	393 (87.7%)	
	Once a week	11 (17.7%)	51 (82.3%)	
	Several times a month	10 (19.2%)	42 (80.8%)	
	Seldom/Never	5 (14.7%)	29 (85.3%)	

Frequency of vegetable consumption	Several times a day	39 (13.2%)	256 (86.8%)	0.575
	Everyday	50 (14.1%)	305 (85.9%)	
	Several times a week	56 (12.3%)	401 (87.7%)	
	Once a week	11 (19.3%)	46 (80.7%)	
	Several times a month	9 (19.1%)	38 (80.9%)	
	Seldom/Never	5 (16.7%)	25 (83.3%)	

Dental visit	Yes	65 (19.9%)	262 (80.1%)	<0.0001^*∗*^
	No	105 (11.5%)	809 (88.5%)	

Mouthwash use	Yes	43 (15.1%)	241 (84.9%)	0.432
	No	127 (13.3%)	830 (86.7%)	

Prosthesis use	Yes	57 (21.0%)	214 (79.0%)	<0.0001^*∗*^
	No	113 (11.6%)	857 (88.4%)	

Diabetes	Yes	50 (24.6%)	153 (75.4%)	<0.0001^*∗*^
	No	120 (11.6%)	918 (88.4%)	

Body mass Index	Underweight	27 (20.1%)	107 (79.9%)	0.002^*∗*^
	Normal	106 (15.0%)	602 (85.0%)	
	Overweight	37 (9.3%)	362 (90.7%)	

^*∗*^ *p* value < 0.05: statistical significant difference.

**Table 5:** Logistic regression analysis with presence of oral potentially malignant disorders as dependent factor.

Factor	Categories	Adjusted Odds ratio	95% Confidence Interval	*p* value
Gender	Male	2.09	1.45–3.00	<0.0001^*∗*^
	Female	Ref		

Tobacco consumption	Ever consumers	2.06	1.07–3.95	0.030^*∗*^
	Never consumers	Ref		

Areca nut chewing	Ever consumers	2.64	1.36–5.13	0.004^*∗*^
	Never consumers	Ref		

Dental visit	Yes	1.03	0.46–2.32	0.939
	No	Ref		

Prosthesis use	Yes	1.16	0.52–2.61	0.720
	No	Ref		

Diabetes	Yes	2.21	1.18–4.16	0.014^*∗*^
	No	Ref		

Body mass Index	Underweight	2.23	1.25–3.99	0.007^*∗*^
	Normal	1.64	1.08–2.49	
	Overweight	Ref		

^*∗*^ *p* value < 0.05: statistical significant difference.
